# Enhanced ferromagnetism of ZnO@Co/Ni hybrid core@shell nanowires grown by electrochemical deposition method

**DOI:** 10.1039/c7ra11123a

**Published:** 2018-01-02

**Authors:** Huyen T. Pham, Tam D. Nguyen, Md. Earul Islam, Dat Q. Tran, Masashi Akabori

**Affiliations:** Japan Advanced Institute of Science and Technology 1-1 Asahidai Nomi 923-1292 Japan s1530048@jaist.ac.jp akabori@jaist.ac.jp; Interdisciplinary Graduate School, Nanyang Technological University Singapore; Energy Research Institute @ Nanyang Technological University Singapore

## Abstract

The hybrid structure of ZnO NWs with the presence of different dopants recently has drawn many interests from researchers due to the possibility to integrate multiple functionalities into one single structure. In this article, we investigated the morphology, crystal structure and ferromagnetism of the ZnO@Co/Ni hybrid core@shell NWs prepared by a facile electrochemical deposition method. The results show that a thin layer of Ni and Co coated on the surface of ZnO NWs (confirmed by XRD, EDS, TEM and Raman scattering) can create a significant improvement of ferromagnetic property in such hybrid core@shell NWs. In which, for the coating time of 10, 15, 20 min, the value of *M*_s_ is around 0.67, 0.88 and 2.56 emu g^−1^ for ZnO@Co NWs, and about 0.013, 0.022 and 0.031 emu g^−1^ for ZnO@Ni NWs, respectively, in comparison with the number of 0.016 emu g^−1^ for pure ZnO NWs. Interestingly, we also found the temperature dependence of ferromagnetism of such Co/Ni coated ZnO NWs. These results reveal the possibility to employ such hybrid core@shell NWs for many applications, *e.g.* spin field effect transistors.

## Abstract 2

Facile electrochemical synthesis of ZnO@Co and ZnO@Ni hybrid core@shell nanowires with enhanced ferromagnetism.

## Graphical abstract

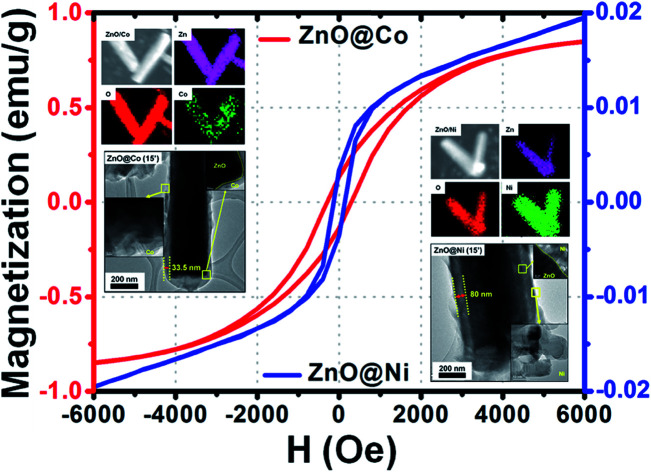

## 1. Introduction

The semiconductor nanowires (NWs), with unique optical, electrical and magnetic features, have been intensively characterized and used in nanoelectronics and nanophotonics applications. Among such materials, ZnO NWs are leading in various energy-related applications mainly in the fields of electronics, spintronics, optoelectronics and photovoltaic devices. ZnO is a semiconductor material with a wide direct band gap of 3.37 eV, high electron mobility, large piezoelectric coefficient, and a large exciton binding energy of 60 meV at room temperature compared to other wide direct band gap materials, *e.g.* GaN (26 meV) or ZnSe (20 meV).^1^ Many different methods can be used to synthesize ZnO NWs, in which, the wet chemical techniques at low temperatures are much more commonly used due to its simplicity, low cost and environmentally friendly. These synthetic methods comprise of chemical bath deposition (CBD),^2–5^ hydrothermal method,^6–8^ and electrochemical deposition method.^9–13^ In particular, electrochemical synthesis strategy is a novel promising technique for fabrication of new heterostructures.

Interestingly, the property of ZnO NWs can be further improved by introducing foreign atoms into the lattice or coating with other materials. Such kinds of hybrid semiconductor nanostructures have drawn many attentions from researchers due to the possibility to combine various functionalities such as electric, optical, photo-catalytic, and magnetic properties. Several different metal and non-metal materials have been used to dope/coat to ZnO NWs, in which, cobalt (Co) and nickel (Ni) are two of the most interesting elements. In the past decades, Co/Ni-doped ZnO nanowires/nanorods (NRs) have been intensively investigated thanks to the simplicity of synthetic process and possibility to control the doping ratio. ZnO NWs with Co/Ni doping have been studied in many different applications, *e.g.* photo-catalysis,^14–18^ spintronics and optoelectronics devices,^19–28^ or sensors…^29,30^ Recently, the researchers have started investigating the property of the Co/Ni coated ZnO NWs in core@shell structure. Fan *et al.* reported the synthesis of well-aligned ZnO@Co hybrid nanotube array by electrochemical deposition method on conductive glass substrates. As-reported vertical-aligned ZnO@Co nanotubes indicate the enhanced photoluminescent properties, improved photocatalytic properties as compared to the bare ZnO NR array, and as well as the ferromagnetism at room temperature.^31^ Filippov *et al.* fabricated ZnO@Ni core@shell NWs using rapid thermal chemical vapor deposition on Au-coated *c*-plane sapphire substrates and investigated by Raman scattering. They observed the change in the structure of ZnO NWs due to the presence of Ni shell, which would be able to utilize in gas sensor application.^32^ In another work, Mudusu *et al.* also presented the fabrication of ZnO NRs by a vapor–liquid–solid method and then coating with Ni nano-layer using an e-beam evaporator. They claimed that the Ni-coated ZnO NRs, with better structural and light emission properties, could be utilized for different device applications, particularly for photoelectrochemical water-splitting devices.^33^ Recently, Deng *et al.* reported the synthesis of ZnO@Ni core@shell NRs using the hydrothermal method, and found the excellent microwave absorption properties of such hybrid nanostructure.^34^

In general, as mentioned reports above mainly inspected the structural, photocatalytic or microwave absorption behavior of Co/Ni coated ZnO NWs. Meanwhile, the intrinsic ferromagnetism of bare ZnO NWs is also an interesting feature which has been studied by several research groups recently, and is promising for many applications of nanoscaled optomagnetics, optoelectronics devices, and biotechnology.^35–38^ Moreover, the synthesis of such hybrid core@shell structure still remains challenging and time-consuming. Therefore, we considered investigating the enhanced ferromagnetism of ZnO NWs by coating with nanolayers of giant positive magnetic Co and Ni materials through a facile synthetic process. The influence of Co/Ni layer thickness and temperatures on the ferromagnetic property of ZnO@Co/Ni core@shell NWs was also investigated. In this article, the ZnO@Co/Ni hybrid core@shell NWs were prepared by a simple electrochemical deposition method. The results show that a thin layer of Ni and Co has been coated on the surface of ZnO NWs (confirmed by XRD, EDS, TEM and Raman scattering), resulting in the improvement of ferromagnetic property in the hybrid core@shell NWs. The changing of electro-deposition time causes to the variation of the magnetic property of such Co/Ni coated ZnO NWs. Interestingly, we also found the temperature dependence of ferromagnetism of such Co/Ni coated ZnO NWs. Moreover, both Co and Ni also have very good electrical conductivity, which is capable of good ohmic contact behavior with ZnO NWs, and therefore suitable for many applications such as field emission transistors.

## 2. Materials and experimental methods

### 2.1 Synthesis of ZnO@Co/Ni hybrid core@shell NWs

**Fig. 1:**
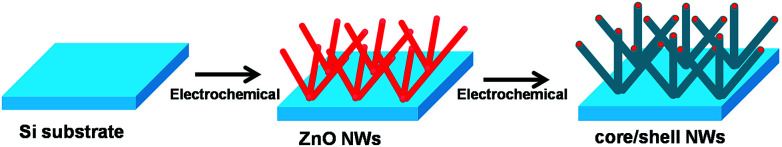
Synthetic scheme for the preparation of ZnO@Co/Ni hybrid core@shell NWs.

The synthetic process of ZnO@Co/Ni hybrid core@shell NWs is shown in Fig. 1. The precursors to grow as-mentioned ZnO@Co/Ni NWs include zinc nitrate hexahydrate (Zn(NO_3_)_2_·6H_2_O, Wako, 99.9%) and hexamethylenetetramine (HMTA, Wako, 99.9%), cobalt(ii) acetate ((CH_3_COO)_2_Co, Wako, 99.9%) and nickel(ii) acetate ((CH_3_COO)_2_Ni·4H_2_O, Wako, 99.9%).

Firstly, the ZnO NWs were electrochemically grown on a p-Si(111) substrate using three electrode system as reported in our previous work.^11^ The p-Si substrate (cleaned and dried under N_2_ flow, then etching in buffered hydrofluoric acid for few seconds to remove the oxide layer) was used as working electrode, while Pt wire and Ag/AgCl were served as counter and reference electrode, respectively. The whole electrode setup was dipped into 100 mL of electrolyte solution composed of 0.025 M Zn(NO_3_)_2_·6H_2_O and 0.025 M hexamethylenetetramine (HMTA) solution at 90 °C. The growth of ZnO NWs was conducted with the applied potential of −0.8 V in 2 h. As-synthesized ZnO NWs afterward were filtrated and washed with acetone, ethanol, DI water and dried in N_2_ flow.

Subsequently, the coating step for such ZnO NWs was conducted to obtain the final the ZnO@Co/Ni hybrid core@shell NWs by dipping as-synthesized ZnO NWs into 0.025 M (CH_3_COO)_2_Co solution or 0.025 M (CH_3_COO)_2_Ni·4H_2_O solution at 70 °C under the applied voltage of −1 V. The electrochemical coating time was varied for 10, 15 and 20 min, respectively, to control the thickness of the magnetic metal coating layer. Finally, ZnO@Co/Ni hybrid core@shell NWs were filtrated and washed with acetone, ethanol, DI water and dried in N_2_ flow.

### 2.2 Characterization

The morphology and elemental composition of as-synthesized ZnO NWs and the ZnO@Co/Ni hybrid core@shell NWs were characterized by scanning electron microscope (SEM, Hitachi S-4500) and energy-dispersive X-ray spectroscopy (EDX) embedded with field emission scanning electron microscopy (FESEM, JEOL 7600F), respectively. A JEOL 2010 transition electron microscope (TEM) was employed to reveal the core@shell structure of as-synthesized NWs. In addition, we studied the crystal structure and lattice parameters of the NWs by X-ray diffraction (XRD, Shimadzu) using Cu-Kα radiation in the range of 20–80° (2*θ* scale). Raman scattering experiments were achieved at room temperature using a conventional and resonance Raman spectroscopy (HORIBA-JY T64000) with the 532 nm line of a semiconductor laser. The ferromagnetic behavior of the NWs was observed using a superconducting quantum interference device (SQUID, Quantum Design MPMS) at different temperatures with the magnetic field from −6000 to 6000 Oe.

## 3. Results and discussion

### 3.1 Morphology and crystal structure characterization

**Fig. 2:**
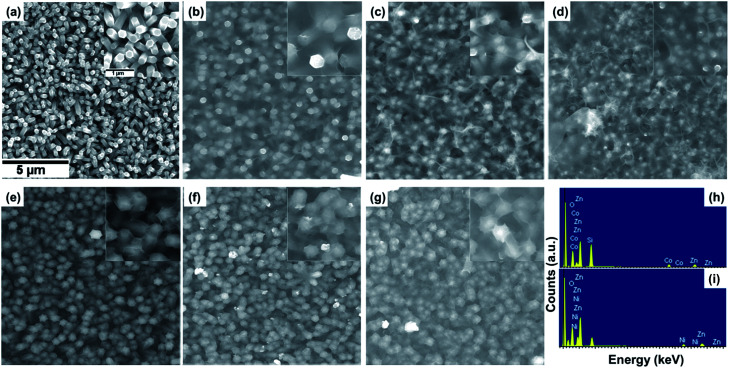
SEM images of ZnO NWs (a), ZnO@Co (b–d) and ZnO@Ni (e–g) hybrid core@shell NWs with electro-deposition time of 10 min (b, e), 15 min (c, f), and 20 min (d, g); EDX spectra of ZnO@Co (h) and ZnO@Ni (i) hybrid core@shell NWs (electro-deposited in 15 min).

The structure and morphology evolution of the ZnO NWs and the ZnO@Co/Ni hybrid core@shell NWs were investigated by SEM. Fig. 2a shows SEM image of as-growth ZnO NWs on Si(111) substrate, revealing their typical hexagonal prism shape, smooth and uniform surface morphology. These NWs consisted of an average diameter, length and density of about 190 nm, 2 μm and 7 μm^−2^, respectively.

**Fig. 3:**
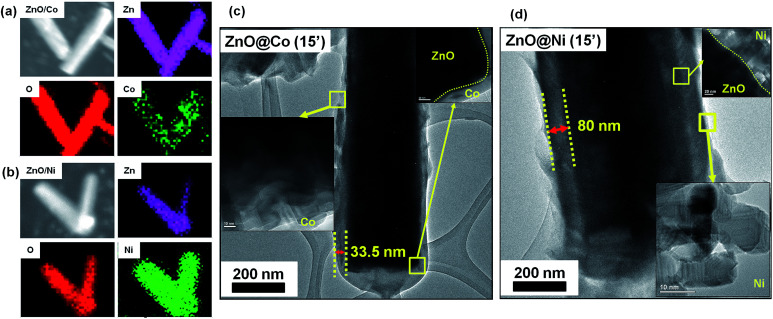
SEM and corresponding elemental mapping images of Zn, O, Ni elements of ZnO@Co (a) and ZnO@Ni (b) hybrid core@shell NWs (electro-deposited in 15 min). TEM images of ZnO@Co (c) and ZnO@Ni (d) hybrid core@shell NWs (electro-deposited in 15 min).

Fig. 2b–d shows the morphology of the ZnO@Co hybrid core@shell NWs on Si(111) substrates with different Co coating times of 10, 15 and 20 min, correspondingly. It can be observed that the Co layer covers the surface of ZnO NWs, and this layer becomes thicker with the increasing coating time. However, this Co layer does not only coat on the ZnO NWs surface, but also forms a thin film on the whole sample substrate. On the tip of the ZnO@Co hybrid core@shell NWs, the Co layers also link together to form a net shape. Fig. 2h shows EDX pattern of the ZnO@Co hybrid core@shell NWs (electro-deposited in 15 min), which reveals that the as-obtained ZnO@Co NWs consist of Zn, O and Co elements. The elemental maps of Zn, O and Co demonstrate that the Co element is evenly distributed on the surface of ZnO and maintains a consistent morphology (Fig. 3a).

For the ZnO@Ni hybrid NWs, Fig. 2e–g indicates the time-dependent shape evolution process of the deposition of Ni on ZnO NWs. It can be observed that the Ni layer covers the outer lateral surface of ZnO NWs after electro-depositing for 10, 15 and 20 min, which are rather rougher as compared to bare ZnO NWs. The Ni layer thickness also increases with the increasing coating time. However, slightly different from Co coating, the Ni layer does not form the thin film on the whole sample substrate, but almost coated on each of single ZnO NW. From EDX pattern of ZnO@Ni hybrid core@shell NWs (electro-deposited in 15 min), it can be concluded that the ZnO@Ni NWs were made up of Zn, O and Ni elements. The elemental maps of Zn, O and Ni prove that the Ni element is evenly distributed on the surface of ZnO and maintains a consistent morphology (Fig. 3b).

The core@shell structure of as-studied ZnO@Co/Ni hybrid NWs is revealed in Fig. 3c and d. It is observed that the Co and Ni layer deposit separately on the ZnO surface, no strange interlayer was detected. The shell thickness was measured to be around 33.5 nm for the ZnO@Co NWs (15 min coating) and about 80 nm for the ZnO@Ni NWs (15 min coating), respectively.

The reaction mechanism of the above ZnO@Co/Ni hybrid core@shell NWs growth process could be described as following:

1 (CH_3_COO)_2_Co → Co^2+^ + 2CH_3_COO^−^

2 Co^2+^ + 2e^−^ → Co

3 (CH_3_COO)_2_Ni·4H_2_O → Ni^2+^ + 2CH_3_COO^−^ + 4H_2_O

4 Ni^2+^ + 2e^−^ → Ni

5 2CH_3_COO^−^ → 2CO_2_ + C_2_H_6_ + 2e^−^

**Fig. 4:**
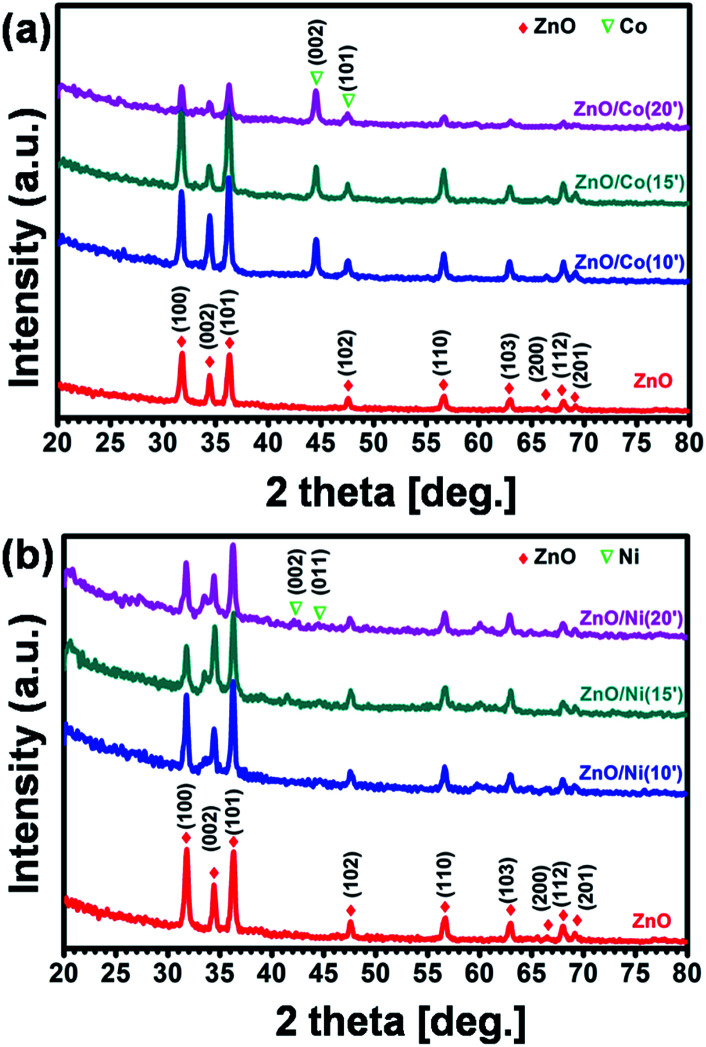
XRD patterns of ZnO@Co (a) and ZnO@Ni (b) hybrid core@shell NWs.

Crystal structures of the synthesized sample were investigated by grazing incidence XRD technique at 1° fixed angle to eliminate the signal of substrates, and the corresponding results are shown in Fig. 4. It is clearly observed the typical diffraction peaks of ZnO NWs at the 2*θ* of 31.5, 34, 36.5, 47.5, 56.5 and 63°, with respect to (100), (002), (101), (102), (110) and (103) plane, correspondingly. All of the diffraction peaks can be indexed to the hexagonal wurtzite structure of ZnO with the lattice parameters of *a* = 3.429 Å and *c* = 5.163 Å, matching with standard data (PDF2 no. 03-065-3411). For ZnO@Co hybrid core@shell NWs, the characteristic peaks at 2*θ* of 44.7 and 47.5° can be assigned to the (002) and (101) plane of Co metal (PDF2 no. 04-003-3863) (Fig. 4a). Fig. 4b indicates XRD patterns of ZnO@Ni hybrid core@shell NWs, the diffraction peaks at 2*θ* of 42.5 and 44.6° are also matched with (002) and (011) crystal surface of Ni metal (PDF2 no. 00-045-1027). Within the detection limit of XRD, no peak characteristic of cobalt or nickel oxide was found in the XRD patterns of the ZnO@Co/Ni hybrid core@shell NWs, correspondingly. These results again conclude the formation of pure Co and Ni shell on the surface of ZnO NWs, which has been showed above.

### 3.2 Raman spectral analysis

**Fig. 5:**
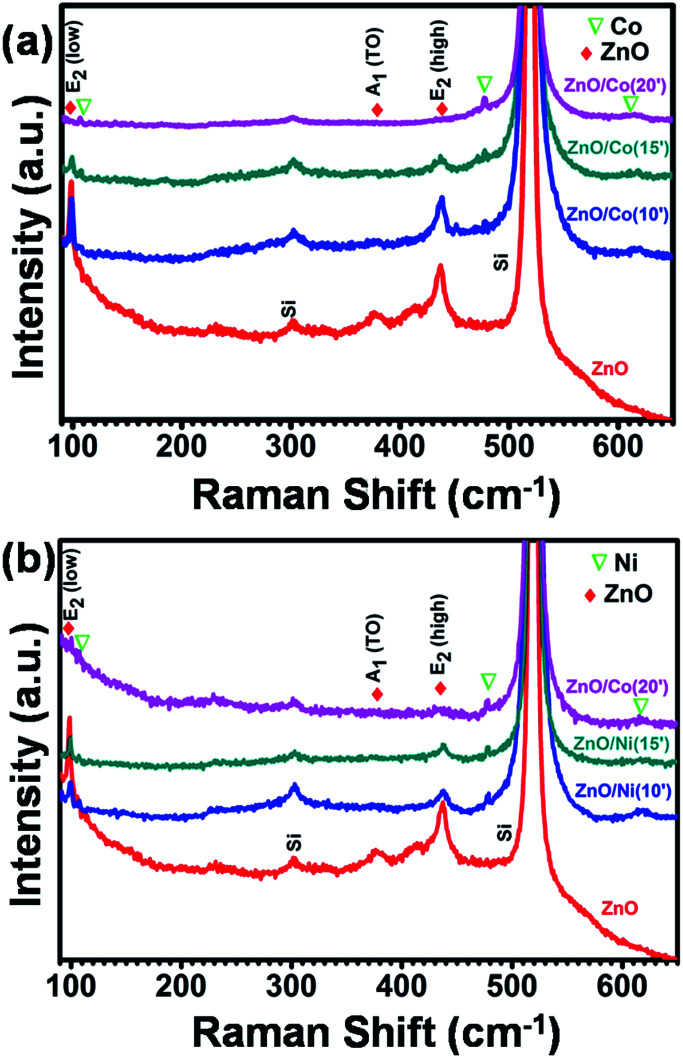
Raman spectra of ZnO@Co (a) and ZnO@Ni (b) hybrid core@shell NWs.

Fig. 5 shows the Raman spectra of pure ZnO NWs, ZnO@Co and ZnO@Ni hybrid core@shell NWs. ZnO indicates *P*6_3_*mc* or *C*_6v_ symmetry and near the center of the Brillouin zone in ZnO, the vibration modes are: A_1_, a doubly degenerate E_1_, two doubly degenerate E_2_ and two B_1_ modes. A_1_ + E_1_ + 2E_2_ are the Raman-active modes in ZnO, where A_1_ and E_1_ are polar, and split into transverse optical (TO) and longitudinal optical (LO) phonons with different frequencies. The peaks of ZnO NWs at 100 cm^−1^ and 439 cm^−1^ were assigned to E_2_ (low) and E_2_ (high), respectively. The second order peak at 332 cm^−1^ was assigned to E_2_ (high)–E_2_ (low) and the peak at 378 cm^−1^ was assigned to A_1_(TO).

We also observed some new peaks at 107, 474 and 618 cm^−1^ in the ZnO@Co hybrid core@shell NWs, which probably belong to the pure Co thin film according to literature (Fig. 5a). In comparison with the study of Zhou *et al.* on the ZnO:Co-doped thin film, we also detected the decline of ZnO peaks and the increment of pure Co peak by the increasing the electro-deposition time of Co.^39^ For instance, by doping with 3, 5, 7, and 12% of Co, the peaks of ZnO at E_2_, A_1_(TO) and A_1_(LO) are gradually dropped. Meanwhile, the Co peaks at 107 and 484 cm^−1^ are rising. All of these variations can be observed in our ZnO@Co hybrid core@shell NWs, with the deposition time of 10, 15 and 20 min. This variation of Raman peaks is attributed to the increasing Co layer thickness with longer deposition time. Fig. 5b shows the Raman spectra of as-growth ZnO@Ni hybrid core@shell NWs. Similar to the analysis of ZnO@Co NWs, we also can see the peaks of Ni at 107.5, 478 and 620 cm^−1^ in the as-synthesized Ni coated ZnO NWs.^40^ With the longer time of Ni electro-deposition, the intensity of ZnO peaks at A_1_(TO) and E_2_ also reduces while the intensity of Ni peaks at corresponding wavelengths slightly increases. This may be also due to the increasing thickness of Ni with longer deposition time.

### 3.3 Ferromagnetism property

The magnetic properties of the ZnO NWs, the ZnO@Co/Ni hybrid core@shell NWs were investigated using a SQUID. The contribution of the Si substrate was subtracted from the raw data.

#### 3.3.1 Magnetic property of ZnO NWs

**Fig. 6:**
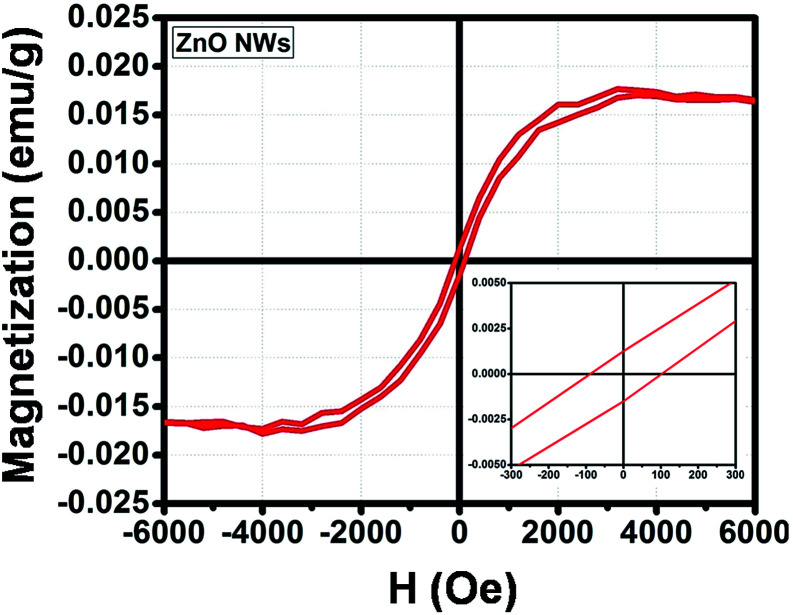
Magnetic hysteresis loop of the ZnO NWs.

Fig. 6 presents the magnified hysteresis (*M*–*H*) curve of ZnO NWs within magnetic field ranging from −6000 to 6000 Oe at room temperature. It was observed that the as-studied ZnO NWs show a ferromagnetic property with the coercivity field (*H*_c_), saturation magnetization (*M*_s_) and the remanence (*M*_r_) of about 100 Oe, 0.016 emu g^−1^ and 0.0016 emu g^−1^, respectively. The observation of ferromagnetism in ZnO NWs is in agreement with several simulation studies for nanoscaled of ZnO material.^35,38,41^ The intrinsic point defect in the structure of ZnO nanomaterials is considered as the responsible factor for its room temperature ferromagnetism. Therefore, it is possible to enhance the magnetic behavior of ZnO NWs, either by increasing the number of intrinsic point defects or doping/coating with other ferromagnetic materials. However, the doping process can also cause to the change in the crystal structure of ZnO NWs, therefore, coating is the most reasonable technique to improve the magnetic property of hybrid ZnO NWs, while maintaining the initial features of the original material.

#### 3.3.2 Magnetic property of ZnO@Co hybrid core@shell NWs

**Fig. 7:**
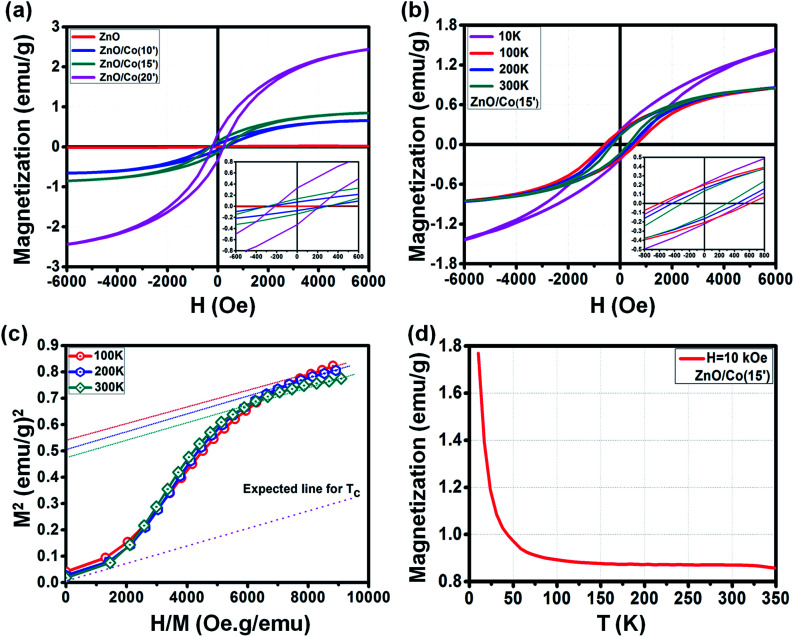
(a) *M*–*H* curve of ZnO@Co hybrid core@shell NWs with different electro-deposition times, (b) *M*–*H* curve of ZnO@Co hybrid core@shell NWs at electro-deposition time of 15 min at different temperatures, (c) the Arrott plot of the *M*–*H* curve of ZnO@Co hybrid core@shell NWs at electro-deposition time of 15 min at different temperatures as shown in (b), (d) *M*–*T* curve of ZnO@Co hybrid core@shell NWs at electro-deposition time of 15 min.

Fig. 7 indicates the ferromagnetic behavior of different ZnO@Co hybrid core@shell NWs. In general, the *M*–*H* curves at room temperature (300 K) show the increasing ferromagnetism of ZnO@Co core@shell NWs with the increment of Co electro-deposition time (Fig. 7a). The detailed values of *H*_c_, *M*_s_ and *M*_r_ for the *M*–*H* curves of as-synthesized ZnO@Co NWs are listed in Table 1. In which, for ZnO@Co hybrid core@shell NWs, the value of *H*_c_ increases up to 290, 300 and 220 Oe for 10, 15 and 20 min coating, respectively, compared to the value of 100 Oe of pure ZnO NWs. The value of *M*_r_ also recorded to be about 0.08, 0.14 and 0.34 emu g^−1^, respectively, for the Co coating time of 10, 15 and 20 min, which are much higher than the value of 0.0016 emu g^−1^ for pure ZnO NWs. Similarly, the value of *M*_s_ for ZnO@Co hybrid core@shell NWs with 10, 15 and 20 min coating times also rises up to 0.67, 0.88 and 2.56 emu g^−1^, correspondingly, while this value for original ZnO NWs is only about 0.016 emu g^−1^.

**Table 1:** The *H*_c_, *M*_s_ and *M*_r_ of ZnO@Co hybrid core@shell NWs at room temperature with different electro-deposition times

Sample@300 K	*H*_c_ (Oe)	*M*_r_ (emu g^−1^)	*M*_s_ (emu g^−1^)
ZnO	100	0.0016	0.016
ZnO@Co(10′)	290	0.08	0.67
ZnO@Co(15′)	300	0.14	0.88
ZnO@Co(20′)	220	0.34	2.56

Interestingly, we observed the variation of the ferromagnetic behavior of such ZnO@Co hybrid core@shell NWs under the effect of temperature (Fig. 7b). By changing the temperature of 10, 100, 200 and 300 K, the modification of *M*–*H* curve for the ZnO@Co core@shell NWs (electro-deposited in 15 min) was detected. The detailed values of *H*_c_, *M*_s_ and *M*_r_ of ZnO@Co core@shell NWs (electro-deposited in 15 min) with various temperatures are listed in Table 2. In detail, at 10, 100, 200 and 300 K, the recorded value for *H*_c_ is about 550, 620, 450 and 300 Oe; for *M*_r_ is about 0.22, 0.2, 0.17 and 0.14 emu g^−1^; and for *M*_s_ is around 1.64, 0.91, 0.89 and 0.88 emu g^−1^, respectively. It is clearly seen that the magnetic behavior of ZnO@Co core@shell NWs (electro-deposited in 15 min) decreases with the increasing temperatures.

**Table 2:** The *H*_c_, *M*_s_ and *M*_r_ of ZnO@Co hybrid core@shell NWs at different temperatures with electro-deposition time of 15 min

Temperature ZnO@Co(15′)	*H*_c_ (Oe)	*M*_r_ (emu g^−1^)	*M*_s_ (emu g^−1^)
10 K	550	0.22	1.64
100 K	620	0.2	0.91
200 K	450	0.17	0.89
300 K	300	0.14	0.88

The Arrott plots for the *M*–*H* curves of ZnO@Co (15′) sample at different temperatures are also presented in Fig. 7c. According to literature, by extrapolating each curve to *M*^2^ = 0, we are able to estimate the Curie temperature (*T*_c_) of ZnO@Co hybrid core@shell NWs.^42,43^ However, as shown in Fig. 7c, the intercept of *H*/*M* axis does not pass through the origin point even at *T* = 300 K, which means that the *T*_c_ of ZnO@Co hybrid core@shell NWs is higher than 300 K. Even though, the expected *T*_c_ line (dash line) is also quite far as compared to the line at 300 K, therefore, we expected that the *T*_c_ of ZnO@Co NWs (15 min) is also very large.

The field cooling (FC) *M*–*T* curve for ZnO@Co hybrid core@shell NWs was also taken at *H* = 10 kOe (Fig. 7d). There is no intersection is found in the temperature range of 10 to 350 K, which again confirms that the *T*_c_ of ZnO@Co hybrid core@shell NWs is higher than 300 K. A study by Pal *et al.* on Co-doped ZnO nanoparticles have claimed the value of *T*_c_ ∼ 800 K for such kind of material.^44^ In addition, the magnetization *M* is strongly increased with the decreasing of temperature *T*, which may imply the contribution of paramagnetic behavior of paramagnetic ions as residuals represented by Curie law, besides the main contribution from pure ferromagnetic Co metal.

#### 3.3.3 Magnetic property of ZnO@Ni hybrid core@shell NWs

**Fig. 8:**
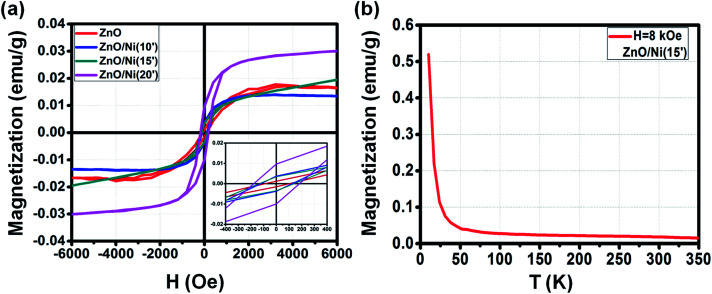
*M*–*H* curve of ZnO@Ni hybrid core@shell NWs with different electro-deposition times (a). *M*–*T* curve of ZnO@Ni hybrid core@shell NWs at electro-deposition time of 15 min (b).

Similar to ZnO@Co NWs, the *M*–*H* curves of different ZnO@Ni hybrid core@shell NWs also reveal its increasing ferromagnetism with respect to the longer Ni electro-deposition time (Fig. 8).

In general, the ferromagnetic property of ZnO@Ni is lower than to ZnO@Co NWs due to the weaker ferromagnetism of Ni in comparison with Co, but still much higher than that of pure ZnO NWs. The detailed values of *H*_c_, *M*_s_ and *M*_r_ for the *M*–*H* curves of as-synthesized ZnO@Ni NWs are listed in Table 3. In detail, the value of *H*_c_ for original ZnO NWs is about 100 Oe, and boosting up to 120, 140 and 180 Oe for 10, 15 and 20 min coating of ZnO@Ni hybrid core@shell NWs, respectively. The value of *M*_r_ is measured to be about 0.04, 0.04, 0.01 emu g^−1^ for ZnO@Ni hybrid core@shell NWs with the electro-deposition time of 10, 15, and 20 min, correspondingly. While the value of *M*_s_ is around 0.013, 0.022 and 0.031 emu g^−1^ for such ZnO@Ni core@shell NWs, correspondingly.

**Table 3:** The *H*_c_, *M*_s_ and *M*_r_ of ZnO@Ni hybrid core@shell NWs at different electro-deposition times and temperatures

Sample@300 K	*H*_c_ (Oe)	*M*_r_ (emu g^−1^)	*M*_s_ (emu g^−1^)
ZnO	100	0.0016	0.016
ZnO@Ni(10′)	120	0.004	0.013
ZnO@Ni(15′)	140	0.004	0.022
ZnO@Ni(20′)	180	0.01	0.031

The field cooling (FC) *M*–*T* curve for ZnO@Ni hybrid core@shell NWs was also taken at *H* = 8 kOe. There is no intersection is found in the temperature range of 10 to 350 K, which again confirms that the *T*_c_ of ZnO@Ni hybrid core@shell NWs is higher than 300 K. The temperature dependence of magnetization *M* is also observed as in the case of ZnO@Co NWs, which also indicates that the magnetic behavior of ZnO@Ni hybrid core@shell NWs is not only originated from the pure ferromagnetic Ni metals but also contributed by the residual paramagnetic ions.

## 4. Conclusions

In this article, we have demonstrated the enhanced ferromagnetism of the ZnO@Co/Ni hybrid core@shell NWs prepared by a facile electrochemical deposition method. By coating a thin layer of Co and Ni on the surface of ZnO NWs (confirmed by XRD, EDS, TEM and Raman scattering), the ferromagnetic property in the hybrid core@shell NWs can be improved. The changing of electro-deposition time causes to the variation of the magnetic property of such Co/Ni coated ZnO NWs. Interestingly, we also found the temperature dependence of ferromagnetism of such Co/Ni coated ZnO NWs. Moreover, both Co and Ni also have very good electrical conductivity, which is capable of good ohmic contact behavior with ZnO NWs, and therefore suitable for many applications such as spin field effect transistors.

## Conflicts of interest

There are no conflicts to declare.

## Supplementary Material
